# Signal Processing for Time Domain Wavelengths of Ultra-Weak FBGs Array in Perimeter Security Monitoring Based on Spark Streaming

**DOI:** 10.3390/s18092937

**Published:** 2018-09-04

**Authors:** Zhenhao Yu, Fang Liu, Yinquan Yuan, Sihan Li, Zhengying Li

**Affiliations:** 1 National Engineering Laboratory for Fiber Optic Sensing Technology, Wuhan University of Technology, Wuhan 430070, China; dwyanesir_yu@whut.edu.cn (Z.Y.); ymyyq@whut.edu.cn (Y.Y.); zhyli@whut.edu.cn (Z.L.); 2 School of Computer Science and Technology, Wuhan University of Technology, Wuhan 430070, China; lsh199395@whut.edu.cn; 3 School of Information Engineering, Wuhan University of Technology, Wuhan 430070, China

**Keywords:** FBGs signal processing, perimeter security monitoring, spark streaming, AP-DBSCAN

## Abstract

To detect perimeter intrusion accurately and quickly, a stream computing technology was used to improve real-time data processing in perimeter intrusion detection systems. Based on the traditional density-based spatial clustering of applications with noise (T-DBSCAN) algorithm, which depends on manual adjustments of neighborhood parameters, an adaptive parameters DBSCAN (AP-DBSCAN) method that can achieve unsupervised calculations was proposed. The proposed AP-DBSCAN method was implemented on a Spark Streaming platform to deal with the problems of data stream collection and real-time analysis, as well as judging and identifying the different types of intrusion. A number of sensing and processing experiments were finished and the experimental data indicated that the proposed AP-DBSCAN method on the Spark Streaming platform exhibited a fine calibration capacity for the adaptive parameters and the same accuracy as the T-DBSCAN method without the artificial setting of neighborhood parameters, in addition to achieving good performances in the perimeter intrusion detection systems.

## 1. Introduction

With the widespread technological development of society, security issues have become increasingly prominent. Thanks to the recent progress of modern science and technology, better solutions have become available to solve security problems. Among them are fiber Bragg grating interference technology [1,2,3], big data processing technology [4,5,6], machine learning [7,8] and stream processing technology [9,10,11]. Recently, our team has realized the online writing of an ultra-weak FBG (UWFBG) array during the drawing process of single mode fibers (SMF). A large-scale UWFBG array is made up of hundreds or thousands of identical-wavelength FBGs with a reflectivity of about −50 dB for each FBG. Such a large-scale UWFBG sensor array has attracted a great deal of attention in major engineering monitoring, because of its low cost, low crosstalk, and strong multiplexing capacity [12,13,14]. In particular, the UWFBGs have the advantages of small size, favorable wavelength selectivity, and anti-electromagnetic interference, and so they are widely used in perimeter security and structural health monitoring.

The physical parameters of these UWFBGs, such as their reflected powers and Bragg wavelengths, vary with external vibration signals. The external signals are extracted from the light signs through demodulation and further data processing. As the demodulated data shows characteristics of having a large capacity, much noise, and a high frequency, it is necessary to detect abnormal data affected by intrusion in a real-time and more accurate way. The outliers can be analyzed to find more abnormal classes by using a clustering algorithm. Thus, how to improve the capability of existing machine learning algorithms to process large-scale data in real-time has become a hot issue.

Handling large-scale data streams requires the support of stream computing [15]. Stream data is a type of data form in the big data environment that was born at the end of 20th century and has gradually become a hot issue in the development of cloud computing and the Internet of Things. Spark Streaming [16] is an extension to the core spark API (application programming interface) [17] that can handle real-time data streams by DStream (discretized Stream). The DStream is expressed by a continuous set of RDDs (resilient distributed datasets) over a time series. Each RDD contains a data stream at a particular time interval. Input data, which is taken from Spark Streaming according to the batch size (such as 1 s), is broken down into segments; each segment is converted to RDD and the results of the RDD operation are stored in the memory. Therefore, Spark Streaming can be used to process large-scale optical fiber grating stream data.

The stream data of perimeter intrusion contains a large amount of information, and the feature parameters of the data are extracted for cluster analysis [18,19,20]. Based on the characteristics of the event types, different clusters can be formed whenever a perimeter intrusion event occurs, which forms a new cluster. More focus should be put on this new type of event in order to detect anomalies in stream data. In the face of unknown distribution data, such as the data collected in perimeter security, the clustering method takes advantage of the relationships among the data objects to gather data in different classes, which is actually an unsupervised way of finding the optimal partition.

The traditional density-based spatial clustering of applications with noise (T-DBSCAN) [21] was proposed by Martin Ester et al. This T-DBSCAN method depends on the choice and calibration of two neighborhood parameters, namely the characteristic size of clusters (*ε*) and the minimum number of points in a cluster (*N*_min_), and so the choice of neighborhood parameters has a great influence on the determination of clusters and it will enable the calibration of a large workload of neighborhood parameters. In recent years, some scholars have tried to improve the T-DBSCAN algorithm through clustering analysis. For example, Li et al., designed a modified DBSCAN to identify fixations in eye-tracking data, thus including the advantages of the classical fixation identification method [22]. Edla et al. proposed a prototype-based modified DBSCAN algorithm to cluster the gene expression data and speed up the DBSCAN algorithm [23]. Cai et al., proposed an improved DBSCAN algorithm that is insensitive to input parameters by adding the connection information of the clusters and merging the related clusters [24], but this method is incapable of selecting neighborhood parameters automatically to achieve unsupervised anomaly detection. Feng et al., proposed an adaptive DBSCAN algorithm for constellation reconstruction and modulation identification [25] by choosing the parameter *N*_min_, and then different values of *ε* were introduced to the trial clustering, the optimal clustering was obtained by evaluating the validity of each cluster. However, it is hard to define the parameter *N*_min_ in the case of trial clustering.

In this paper, an adaptive-parameters DBSCAN (AP-DBSCAN) method was proposed, in which two neighborhood parameters, *ε* and *N*_min_, were determined automatically by time-domain statistical analysis. Then, AP-DBSCAN was implemented on Spark Streaming to deal with the problems of data stream collection and real-time analysis. After that, a perimeter intrusion detection system based on suspended UWFBG sensing arrays and UWFBG sensing arrays that were buried under the ground was constructed. Finally, a number of sensing and processing experiments were finished and analyzed.

## 2. Materials and Methods

### 2.1. T-DBSCAN

DBSCAN is a famous density based clustering technique [21]. A cluster in this model is described as a linked region that exceeds a given density threshold. The functioning of DBSCAN is directed by two definitions, namely density-reachability and density-connectability, which depend on two predefined parameter values: the size of the neighborhood, denoted by *ε*, and the number of neighborhood points in a cluster, denoted by *N*_min_. In T-DBSCAN, one begins with a random point x and it finds all of the points that are density-reachable from x with respect to *ε* and *N*_min_. It is obvious that no points are density-reachable from x when x is a border point; in this case, the T-DBSCAN begins with an unclassified point to repeat the same process, and so the two predefined parameters *ε* and *N*_min_ decide the quality and efficiency of clusters.

### 2.2. AP-DBSCAN Algorithm

In the T-DBSCAN method, the values of *ε* and *N*_min_ are regulated by the users. To avoid human intervention, we proposed an unsupervised clustering method. A sample set composed of *n* sensing signals, ${\mathrm{Signs}}_{n}=\left\{\left(x_{l}\right),l=1,2,\ldots ,n\right\}$, with the sampling frequency (*f*) and a quantity of *n_f_* = *n*/*f*, can be denoted by

(1) $${\mathrm{Signs}\;}_{n}=\left\{\left(s_{m}\right),m=0,1,2,\ldots ,n_{f}\right\}$$

where

(2) $$s_{m}=\left\{x_{mf+1\;},x_{mf+2},\ldots ,x_{mf+f}\right\}$$

Then, a set of characteristic parameters, energy and average amplitude, can be calculated, as follows:

(3) $$energy=\left\{\sum_{i=1\;}^{f}x_{mf+i}^{2},m=0,1,2,\ldots ,n_{f}\right\}$$

(4) $$average\;amplitude=\left\{\frac{1}{f}\sum_{i=1\;}^{f}\left|x_{mf+i}\right|,m=0,1,2,\ldots ,n_{f}\right\}$$

A set of sample characteristic parameters can be described as:

(5) $$T=\left\{\left(\sum_{i=1}^{f}x_{i}^{2},\frac{1}{f}\sum_{i=1}^{f}\left|x_{i}\right|\right),\left(\sum_{i=1}^{f}x_{i+f}^{2},\frac{1}{f}\sum_{i=1}^{f}\left|x_{i+f}\right|\right),\left(\sum_{i=1}^{f}x_{i+2f}^{2},\frac{1}{f}\sum_{i=1}^{f}\left|x_{i+2f}\right|\right),\ldots ,\left(\sum_{i=1}^{f}x_{i+n_{f}f}^{2},\frac{1}{f}\sum_{i=1}^{f}\left|x_{i+n_{f}f}\right|\right)\right\}$$

These characteristic data contain different dimensions. An effective normalization is needed to eliminate the influence of target dimensions, and so the min-max normalization method was used, which enables the mapping of energy and average amplitude in the range of [0,1] by the linear transformation of the characteristic set T. Let the horizontal axis and vertical axis in the range of [0,1] denote normalized energy and normalized average amplitude, respectively; then, the original one-dimensional data were converted into two-dimensional normalized data. Then, a symmetric distance matrix which describes the distances between all pairs of points may be constructed, as follows:

(6) $$D_{t}=\left[\begin{array}{cccc} d_{11} & d_{12} & \ldots & d_{1t}\\ d_{21} & d_{22} & \ldots & d_{2t}\\ \vdots & \vdots & \ddots & \vdots \\ d_{t1} & d_{t2} & \ldots & d_{tt} \end{array}\right]$$

where *t* *=* 1 *+ n/f* is the number of the characteristic sample sets and *d_ij_* is the distance between points *i* and *j*.

Sorting the elements of each row in the matrix **D_t_** from small to large in turn, a new matrix **D_s_** can be obtained. In the matrix **D_s_**, all of the elements at the first column are zero, and the elements at the kth column (k > 1) are the (k − 1)^th^ closer distances. The sorted matrix **D_s_** can be represented by column matrices:

(7) $$D_{s}=\left(\zeta_{1},\zeta_{2},\ldots ,\zeta_{t}\;\right),\;\zeta_{i}={\left(d_{1i},d_{2i},\ldots ,d_{ti}\right)}^{T}$$

For all the column matrices, calculating their *J* values gives the following:

(8) $$J_{i}=J\left(d_{1i\;},d_{2i},\ldots ,d_{ti}\right)=\frac{1}{2}{\left(\sum_{i=1}^{t}d_{1i}-\frac{1}{t}\sum_{i=1}^{t}d_{1i}\right)}^{2}\;i=1,2,\ldots ,t$$

Then, to find the characteristic column matrix, which produces a minimum of all *J*’s values, this process can be denoted by:

(9) $$\gamma =\arg\min\left(J\left(\zeta_{i}\;\right),i=1,2,\ldots ,t\right)$$

Thus, the characteristic column matrix $\gamma =\zeta_{i\min}={\left(d_{1,i\min},d_{2,i\min},\ldots ,d_{t,i\min}\right)}^{T}$ can be obtained. Progressively, the maximum distance in the characteristic column matrix was assigned as *ε*.

After the determination of *ε*, and then performing an arithmetic mean for the number of points within the *ε*-neighborhood in the entire data set, an optimal value of the point number in each cluster can be obtained:

(10) $$N_{\min}=\frac{1}{n}\sum_{i=1}^{n}X_{i}$$

where *X_i_* is the number of points in the *ε*-neighborhood of each point.

### 2.3. AP-DBSCAN on Spark Streaming

**Figure 1:**
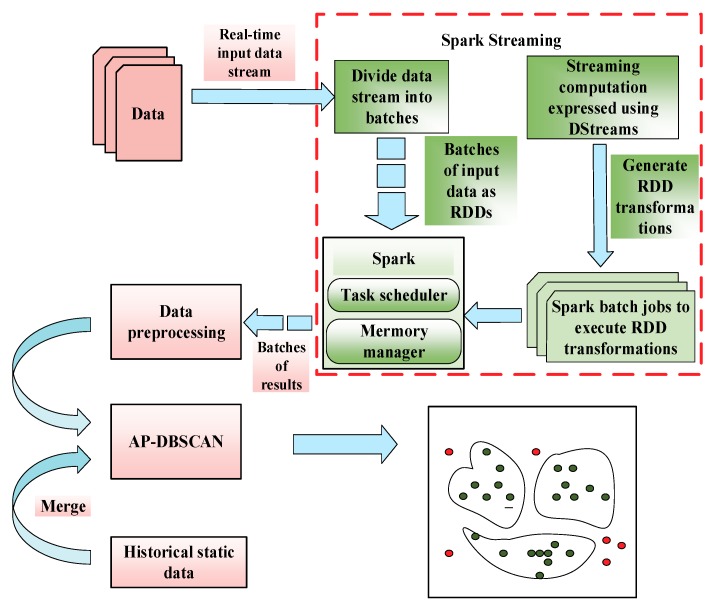
Adaptive parameters density-based spatial clustering of applications with noise (AP-DBSCAN) clustering analysis based on the Spark Streaming mechanism.

Figure 1 describes the implementation process of the proposed AP-DBSCAN method on Spark Streaming, which decomposes streaming computing into a series of short batch jobs. The batch engine is Spark Core, which divides the input data into pieces of data according to the batch size (for example, 4 s). The data were converted to the RDD in Spark, and then the transformation operation was changed to the RDD transformation operation, and each RDD is the data conversion of T s demodulated by the fiber grating signal processor.

**Figure 2:**
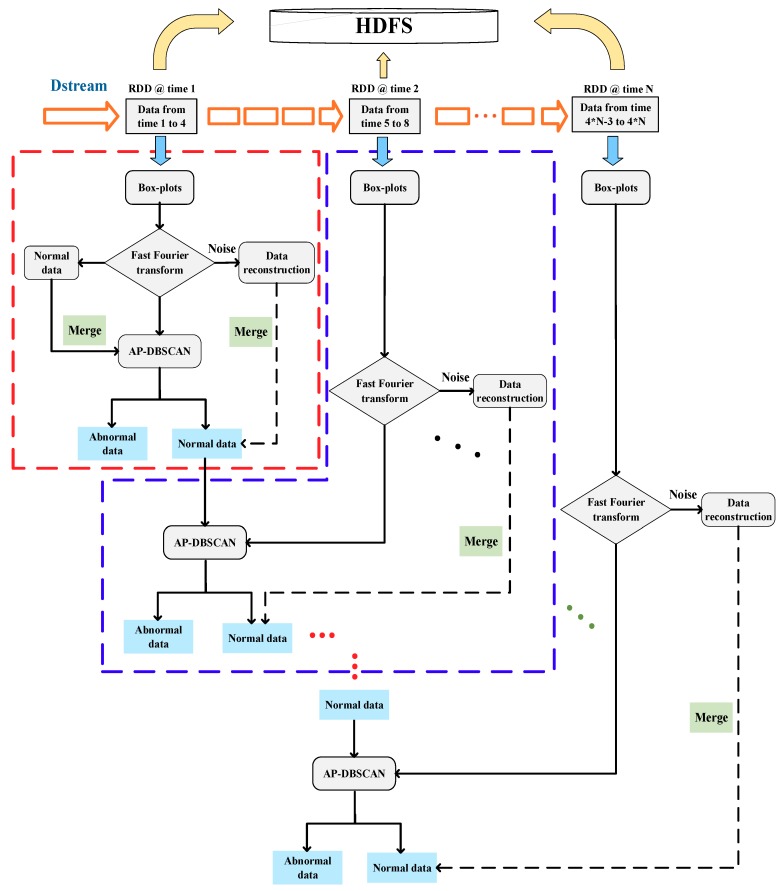
The workflows of AP-DBSCAN implementation on Spark Streaming.

The main steps in AP-DBSCAN on Spark Streaming are shown in Algorithm 1, and the workflows of the algorithm are shown in Figure 2. Box-plots [26] and fast Fourier transform [27] are employed to deal with noises. The feature sets of normal data in the first RDD, which mean that there is no intrusion, are obtained by Equations (3) and (4), and are then mixed to the proposed AP-DBSCAN in case the abnormal data appears at the beginning. The noise data is reconstructed and added to the normal data from AP-DBSCAN. On the DStream, each piece of data is continuously updated by RDD. If there is no abnormal data in a certain RDD, the output of the RDD includes normal feature samples of the last RDD and the current RDD; if there is abnormal data in a certain RDD, then the output of the RDD includes not only the normal feature samples of the last RDD and the current RDD, but also the abnormal samples of the current RDD. Thus, the clustering result of each RDD is achieved through AP-DBSCAN when the distinguished abnormal samples are output and the normal data samples are mixed with the following RDD.

**Algorithm 1.** Main steps in AP-DBSCAN on Spark Streaming.1: Input:  The training sets of *n* workers: $D_{n^{}}^{}=\left\{\left(x_{1}^{},y_{1}^{}\right),\left(x_{2}^{},y_{2}^{}\right),\ldots ,\left(x_{n^{}}^{},y_{n^{}}^{}\right)\right\}$ Normal data: $N_{t}=\left\{\left(x_{1},y_{1}\right),\left(x_{2},y_{2}\right),\ldots ,\left(x_{t},y_{t}\right)\right\}$2: Step1: Create a local streaming context with two working thread and a batch interval of 4 s.3: Step2: Create an input in DStream.4: Step3: Operate DStream:  Convert segment data and normal data to RDD, perform the first AP-DBSCAN to get the result of the clustering: $\;D_{i}:\{\left(x_{1},y_{1}\right),\left(x_{2},y_{2}\right),\ldots ,\left(x_{i},y_{i}\right)\}\cup N_{t}:\left\{\left(x_{1},y_{1}\right),\left(x_{2},y_{2}\right),\ldots ,\left(x_{t},y_{t}\right)\right\}$    →first RDD→AP-DBSCAN→the first clustering result  While input DStream = true  Abnormal data is separated from the first result, normal data is retained and mixed into the next data;  Perform AP-DBSCAN to get the result of clustering.5: Step4: Start Spark Streaming.6: Output: The results of clustering on each RDD.

## 3. Results and Analysis

### 3.1. Monitoring System Based on the UWFBG Array

**Figure 3:**
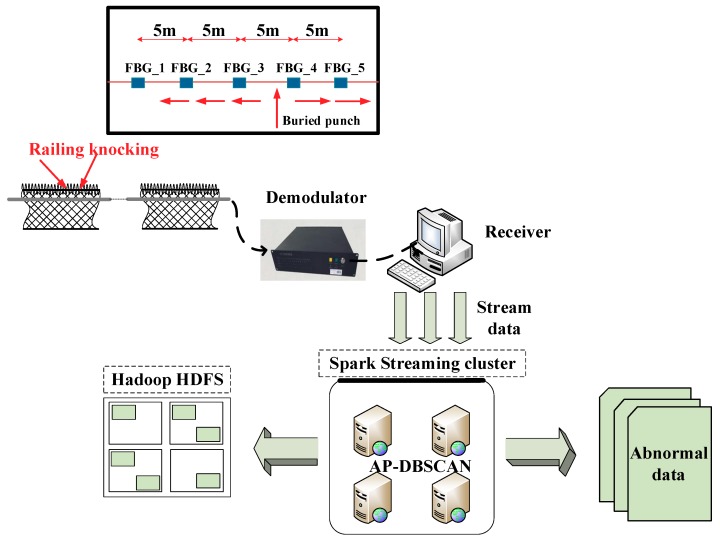
Architecture of the intrusion monitoring and identification system.

The architecture of the intrusion monitoring and identification system is shown in Figure 3. The sensing system is composed of a quasi-distribution UWFBG array that was prepared on a drawing single mode silica optical fiber, a 1550-nm laser source (RIO, narrow frequency laser module, 1 kHz of line width), an FBG signal processor, a detector (4-way photoelectric detection plate, self-control, bandwidth is 60 MHz), and a computer. UWFBGs with the same Bragg wavelengths were used as a string of vibration detectors to encapsulate the external vibration signals near the optical fibers. In our experiments, two kinds of UWFBG sensing arrays were prepared at a distance of every 5 m: one is the suspended UWFBG sensing array with a length of 100 m (20 sensors), which was fixed along a railing; the other is the buried UWFBG sensing array with a length of 300 m (60 sensors), which was buried under the ground.

All of the wavelength shift signals from the UWFBGs were transmitted to the signal processor based on Mach-Zehnder interference (MZI). The grating signal processor was connected to a computer through a network line, sending and receiving the data by the user datagram protocol (UDP). The computer and the software received the data stream from the grating signal processor, pushed the stream data to Spark Streaming for real-time processing, and saved the data to the Hadoop distributed file system (HDFS). As abnormal data appeared, the computer output them in real-time, and finally actualized the intelligent analysis and pattern recognition of the intrusion signals.

The data acquisition methods are as follows: the continuous light from an amplified spontaneous emission (ASE) was modulated into a nanosecond pulse by a semiconductor optical amplifier. The pulse light was launched into the fiber with uniformly distributed UWFBGs by a circulator, and then a pulse train could be realized. A phase demodulation unit consisting of an unbalanced MZI, a 3 × 3 coupler and three detectors was used to restore the vibration signal. The unbalanced paths of the MZI separated each reflected pulse to two pulses; the slower pulse from the closer UWFBGs coincided with the faster pulse from the further UWFBGs, and the coherence would be maintained. Phase perturbations that are caused by vibrations between the two adjacent UWFBGs can be demodulated from the interference light pulse. According to optical time domain reflectometry, the correspondence relationships between the interference light pulse and sensing position were established.

The experiments were finished on a cluster with four nodes: a master node and three compute nodes. The configuration of each node is as follow: 3.4 GHz Intel Core i7-6700 processor, 8 M cache, 4 G memory, and 1 TB storage. The software used are Spark 1.6.1 and the Ubuntu 16.04 operating system, and the sampling frequency is 100 Hz.

### 3.2. Signal Processing for Railing Sensors

**Figure 4:**
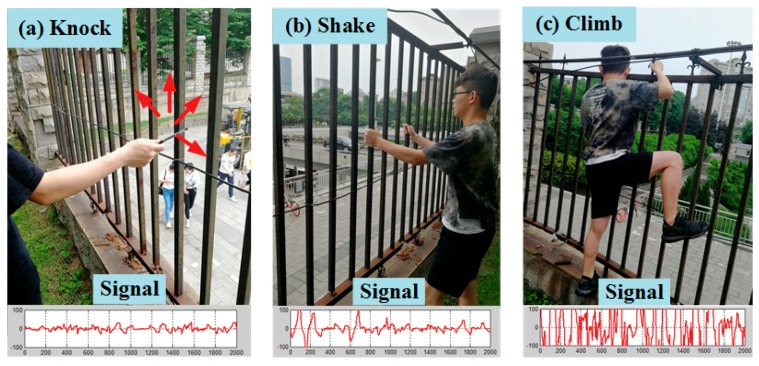
Three types of railing intrusion. (**a**) Knocking the railing; (**b**) shaking the railing; and, (**c**) climbing on the railing.

**Figure 5:**
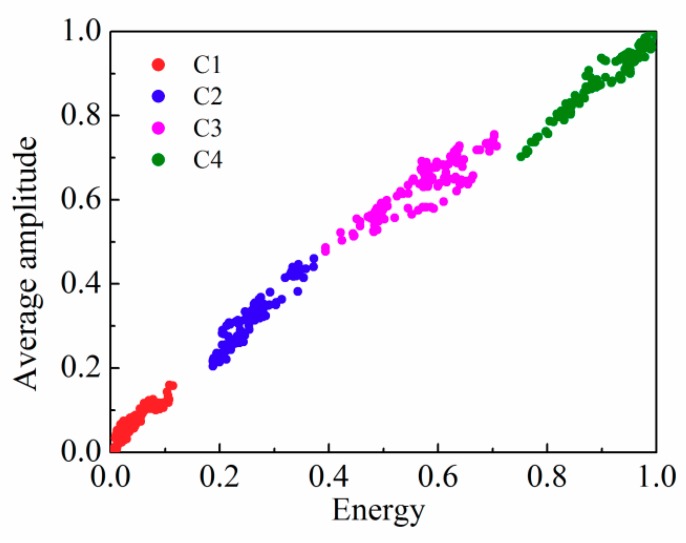
AP-DBSCAN results of three kinds of railing intrusion behaviors.

Three kinds of railing intrusion behaviors, namely knock, shake, and climb, were simulated, and the corresponding vibration signals are shown in Figure 4a–c. The DBCSAN and AP-DBSCAN calculations were finished by selecting the data of three behaviors and merging static data, respectively. The corresponding clustering effects are shown in Figure 5, where the horizontal axis and vertical axis denote the normalized energy and the normalized average amplitude, respectively. According to the distance between the mass center of each cluster and the origin, one can judge the type of behaviors and each cluster represents a behavior.

In addition, the number of points in each cluster and the number of clusters corresponding to all the behaviors given by the T-DBSCAN and AP-DBSCAN methods were calculated and are shown in Table 1. The number of data for static, knocking, shaking, and climbing the rail are represented by C1, C2, C3, and C4, respectively; the results showed that the AP-DBSCAN method can achieve the same precision as T-DBSCAN without setting neighborhood parameters manually, being conductive to the realization of automatic detection in the perimeter intrusion detection systems.

Finally, according to the methods by K-means [28], FCM (Fuzzy C-means) [29], and AP-DBSCAN, the misclassified patterns, the computation time and the error rate (ER) were computed and compared; their data are shown in Table 2, where the error rate was computed, as follows:

(11) $$ER=\frac{Number\;of\;misclassified\;objects\;}{Total\;number\;of\;objects}\times 100\%$$

Smaller ERs given by AP-DBSCAN indicated the AP-DBSCAN method can produce better results than the other methods.

### 3.3. Signal Processing for Buried Sensors

**Figure 6:**
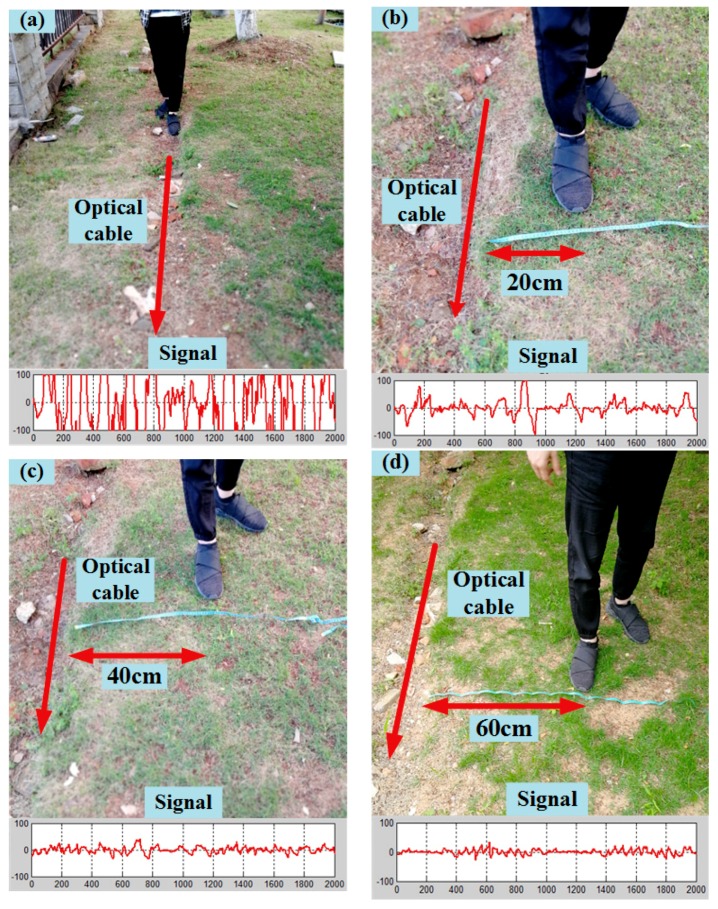
Four types of underground fence intrusion. (**a**) Walking on the buried cable; (**b**) walking parallel to the cable at a distance of 20 cm; (**c**) walking parallel to the cable at a distance of 40 cm; and, (**d**) walking parallel to the cable at a distance of 60 cm.

**Figure 7:**
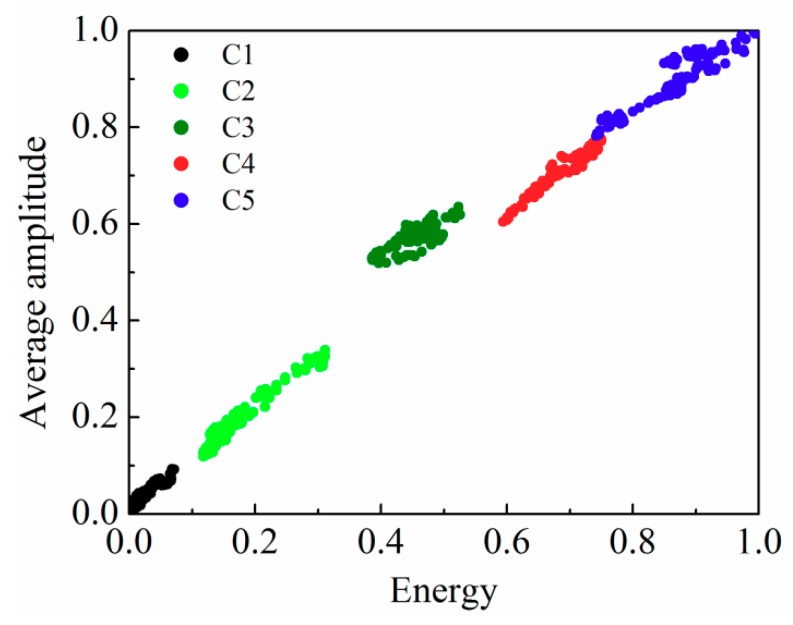
AP-DBSCAN results of four kinds of buried intrusion behaviors.

The underground sensing optical cable was buried under the ground at a depth of half a meter so that the optical cables were less affected by noise. We simulated five behaviors that influence the underground cable: walking on the buried cable, walking parallel to the cable at distances of 20, 40, 60 cm from the cable, and static standing, respectively. Each behavior records data for 20 s, and the corresponding vibration signals are shown in Figure 6a–d. Due to low noise, the collected data is processed by difference denoising methods, and then the AP-DBSCAN method is used to calculate the clustering based on the data of five behaviors. The corresponding clustering effects are shown in Figure 7, where the horizontal axis and vertical axis denote the normalized energy and the normalized average amplitude, respectively. The AP-DBSCAN on Spark Streaming and that on a single machine are compared with the clustering speed at an interval of 4 s. The abnormal behavior is obtained by clustering according to the data characteristics of different behaviors. Each cluster represents one kind of behavior; the abscissa and the ordinate of point denote energy and frequency amplitude, respectively. According to the distance from the origin to the center of the cluster, one can determine the type of the behaviors.

Then, we calculate the number of clusters that form each behavior and the number of points in each cluster, as given by T-DBSCAN and AP-DBSCAN. The number of data sets of static standing, walking parallel to the cable at distances of 60, 40, and 20 cm from the cable, and walking on the buried cable is denoted by C1, C2, C3, C4, and C5, respectively. According to the same sample data, the experimental results of the two methods are shown in Table 3, which exhibits that the AP-DBSCAN method can achieve the same accuracy as the T-DBSCAN without artificial setting of neighborhood parameters; thus, the AP-DBSCAN method can save a large amount of labor time.

Thirdly, according to the methods by K-means [28], FCM (Fuzzy C-means) [29], and AP-DBSCAN, the misclassified patterns, the computation time, and the error rate (ER) were computed and compared; their data are shown in Table 4. One can see that the smaller ERs given by AP-DBSCAN indicated that the AP-DBSCAN method can produce better results than the other methods.

**Figure 8:**
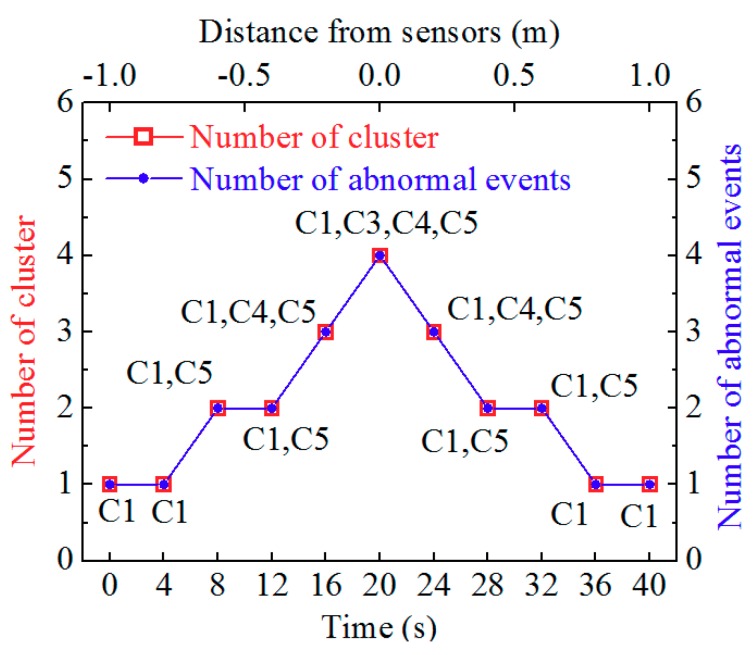
Time response of AP-DBSCAN on Spark Streaming.

**Figure 9:**
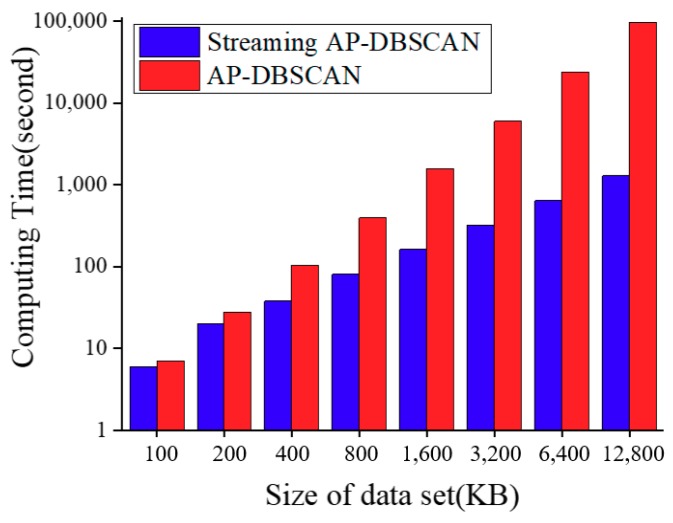
Computing times by AP-DBSCAN and AP-DBSCAN on Spark Streaming.

Finally, the time response of AP-DBSCAN on Spark Streaming was investigated also. In order to test the timeliness of the algorithm, several invasion scenarios were simulated. At a distance of 1 m from one side of the detection optical cable, one person walked to the optical cable vertically and arrived at the ground over the optical cable, and then walked 1 m distance to other side of the optical cable. Meanwhile, 100 pieces of data are recorded every 1 s and calculated every 2 s; the clustering data and the number of abnormal events are shown in Figure 8. It can be seen that the data can be received and processed in an effective time. To test the performance of the AP-DBSCAN method on Spark Streaming, the time responses was measured and shown in Figure 9. There is a small difference between AP-DBSCAN on a single machine and AP-DBSCAN on Spark Streaming when the test data is small. However, when the test data is very big, the response time by the AP-DBSCAN on Spark Streaming is significantly superior to the response time by AP-DBSCAN on the single machine.

## 4. Conclusions

In this paper, we propose the AP-DBSCAN algorithm with adaptive parameters on the Spark Streaming platform, solving the problem of the real-time anomaly detection of large-scale data in perimeter security. The preprocessing of the algorithm combines the Box-plots and the fast Fourier transform, and it is necessary to make certain that the data stream of a segment is mixed with the normal data stream of the previous segment to detect the abnormal data of different types. In the verification experiment of AP-DBSCAN, the proposed algorithm improves the unsupervised capability of T-DBSCAN and can detect abnormal conditions of large-scale data in real-time, providing better convenience and service for perimeter security.

## Figures and Tables

**Table 1:** Comparison of calculated data by two methods for railing sensors.

Data Set	Clustering Algorithm	
	T-DBSCAN	AP-DBSCAN
C1	119	119
C2	113	113
C3	111	111
C4	121	121
Number of clusters	4	4

**Table 2:** Comparisons of misclassified patterns, computation time and the error rate (ER) for different sizes of data sets for railing sensors. A: K-means; B: Fuzzy C-means (FCM); C: AP-DBSCAN.

Data Size (kB)	Misclassified Patterns (kB)			Computation Time (s)			ER (%)		
	A	B	C	A	B	C	A	B	C
150	12	3	**3**	10	10	**11**	8.0	2.0	**2.0**
185	15	4	**5**	27	29	**25**	8.1	2.1	**3.0**
286	26	10	**9**	43	44	**41**	9.1	3.5	**3.1**
768	59	24	**21**	391	401	**379**	7.7	3.1	**2.8**
1024	93	32	**33**	578	593	**533**	9.1	3.2	**3.3**
1625	131	49	**47**	1601	1701	**1567**	8.1	3.0	**3.0**

**Table 3:** Comparison of calculated data by two methods for buried sensors.

Data Set	Clustering Algorithm	
	T-DBSCAN	AP-DBSCAN
C1	116	116
C2	115	115
C3	115	115
C4	115	115
C5	114	114
Number of clusters	5	5

**Table 4:** Comparisons of misclassified patterns, computation time and the error rate (ER) for different sizes of data sets for buried sensors. A: K-means; B: FCM; C: AP-DBSCAN.

Data Size (kB)	Misclassified Patterns (kB)			Computation Time (s)			ER (%)		
	A	B	C	A	B	C	A	B	C
131	10	5	**2**	9	9	**10**	7.6	3.8	**1.5**
254	23	15	**7**	40	42	**37**	9.1	5.9	**2.8**
552	46	27	**13**	287	301	**266**	8.3	4.9	**2.4**
783	62	34	**23**	399	420	**391**	7.9	4.3	**2.9**
1131	101	81	**39**	583	606	**542**	8.9	7.1	**3.4**
1721	140	121	**51**	1721	1835	**1643**	8.1	7.0	**3.0**
